# Protective role of baicalin against methylparaben-induced reproductive toxicity: Insights into hormonal and enzymatic regulation

**DOI:** 10.1016/j.toxrep.2025.102113

**Published:** 2025-08-15

**Authors:** Daniel Itiza Akaahan, Augustine Uche Agu, Nkemjika Chinyere Anyanwu, Vivian Onyinye Orjiako, Godson Emeka Anyanwu

**Affiliations:** a Department of Anatomy, Faculty of Basic Medical Sciences, College of Medicine, University of Nigeria Enugu Campus, Enugu, Nigeria; b Department of Microbiology and Immunology, Faculty of Biomedical Sciences, College of Medicine, Kampala International University, Uganda; c Department of Anatomy, Faculty of Biomedical Sciences, College of Medicine, Kampala International University, Uganda

**Keywords:** Methylparaben, FSH, LH, Testosterone, Reproductive toxicity, 17β-hydroxysteroid dehydrogenase 3 (17β-HSD3)

## Abstract

**Background:**

Methylparaben is a commonly used preservative in the cosmetics, pharmaceutical, and food industries, valued for its antibacterial and antifungal effects. Numerous in vitro and in vivo studies have investigated its adverse effects on sperm count, testosterone levels, and reproductive organ weight. Baicalin, which comes from the dried roots of the plant Scutellaria baicalensis Georgi, is a natural compound that may have various health benefits, such as reducing fibrosis, itching, bacteria, oxidative stress, inflammation, and cancer. This study investigated the effect of baicalin on the changes in male reproductive hormones and enzyme activity brought about by methylparaben.

**Method:**

A total of forty-five adult male Wistar rats were randomly allocated into nine distinct groups, each comprising five rats. Over a period of 28 days, these subjects were administered treatments via gastric gavage, which included distilled water, peanut oil, methylparaben, or differing doses of baicalin, either in isolation or in conjunction with methylparaben. Post-treatment, blood samples were obtained under terminal anesthesia for the purpose of serum analysis. Assays for hormonal levels (FSH, LH, testosterone) and enzymatic activity (17β-HSD3) were performed utilising ELISA and spectrophotometric techniques in accordance with established protocols.

**Results:**

Rats treated with methylparaben (Group 3) had much lower levels of FSH, LH, testosterone, and 17β-HSD compared to the other groups, and baicalin was able to reduce these effects in a dose-dependent manner. Higher baicalin doses restored hormone and enzyme levels to near-control values levels indicating its protective benefits.

**Conclusion:**

The results indicate that baicalin could mitigate the reproductive toxicity induced by methylparaben, owing to its antioxidant and regulatory characteristics, highlighting its potential as a protective agent against endocrine-disrupting chemicals

## Highlights

• Methylparaben was found to significantly reduce serum FSH, LH, testosterone and 17β-HSD3 levels in male Wistar rats.
• Baicalin co-administration restored dose-dependent hormonal and enzymatic disruptions produced by methylparaben.
• Baicalin's antioxidant and endocrine-regulating capabilities likely mediate its protective effect.
• High-dose baicalin significantly restored reproductive hormone and enzyme levels to almost normal values.
• Research suggests enormous potential for Baicalin as a natural treatment for endocrine-disrupting chemical exposure.

## Graphical Abstract

**Figure:**
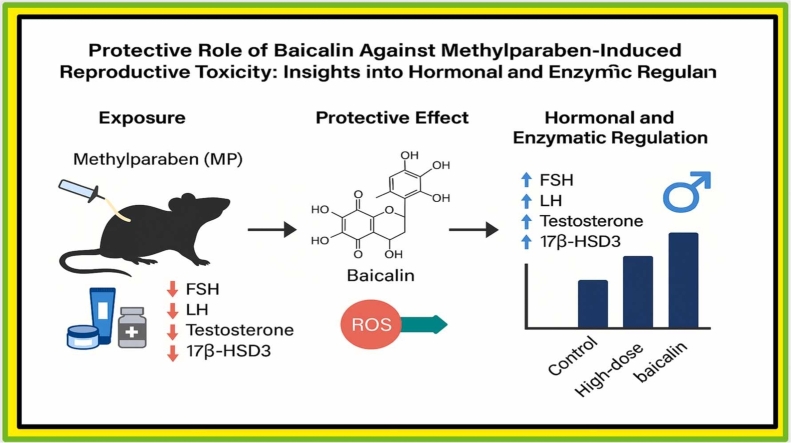

## 1 Introduction

A variety of compounds prevalent in the environment, such as cadmium, lead, and bisphenol A, have been recognised as endocrine disruptors due to their ability to interfere with hormonal signaling pathways. The effects of these substances are mediated through various mechanisms, including oxidative stress, receptor antagonism, and the disruption of the hypothalamic-pituitary-gonadal axis, culminating in reproductive toxicity and hormonal changes [1], [2], [3], [4], [5], [6], [7]. Methyl paraben (CAS No. 99-76-3) is the methyl ester of p-hydroxybenzoic acid. For over five decades, this non-volatile, stable compound has served as an antimicrobial preservative in foods, drugs, and cosmetics [8]. It belongs to a family of parabens that can be used independently or in combination to achieve the desired antimicrobial effect.

Methylparaben is a colorless crystalline or white powder, usually odorless or with a faint characteristic odor and a mild burning flavor, appearing in its purest form. It is hydrolyzed invariantly in hot as well as cold water and in an alkaline solution. It can be synthesized by esterifying p-hydroxybenzoic acid with methanol in sulfuric acid [9], and methylparaben is an air stable substance. It is commonly used as a preservative in the pharmaceutical and food industries as well as in cosmetics for its antibacterial and antifungal effects [10]. It is the most commonly used preservative in personal care products [11]. Though it is considered potentially harmful to human health, it is suspected to be an endocrine disruptor – an agent that might interfere with normal hormonal function [12].

Over the past few decades, parabens' effects on oxidative stress and infertility have been studied extensively in vitro and in vivo in animal models. Subsequently, many of these studies have been focused on male rodent infertility and they indicated that parabens caused a drop in the sperm count and the testosterone levels along with the weight of reproductive organs on mice infected with them in their diet [13]. The induction of oxidative stress by parabens has been clearly demonstrated, with even the least toxic paraben, methylparaben (MP), contributing to the production of glutathione hydroquinone and glutathione-benzoquinone conjugates through reactions with oxygen singlet (1O2) and glutathione (GSH), as well as the production of hydrogen peroxide [14]. Long-term exposure to low levels of xenoestrogens (XEs) has been implicated in the increased incidence of hormone-dependent diseases. Methylparaben can induce oxidative stress [15], cause significant delays in developmental times and decrease fecundity [16], disrupt estrogenic and androgenic receptors [17], [18], [19], and impair sperm motility and quality [13], [20].

Natural compounds have been reported to have a better ability to enhance reproductive hormone regulation as well as hypothalamic-pituitary gonadal (HPG) axis activity in preclinical models [21], [22], [23], and it serves to justify research on natural compounds such as baicalin to treat endocrine disturbances. The bioactive flavonoid baicalin isolated from Scutellariae Radix (the dried roots of Scutellaria baicalensis Georgi) has demonstrated multiple potential pharmacological activities including anti-fibrotic, anti-pruritic anti-bacterial, anti-oxidant, anti-inflammatory and anti-cancer effects [24]. Baicalin, as its name implies, is a light yellow powder with a bitter taste, and has typical solubility properties, such as insolubility in the alcohols and solubility in chloroform, nitrobenzene, dimethyl sulfoxide, etc [25], [26]. Its differential neuroprotective ability has been showed in the treatment of various diseases [27], [28].

While there are reports on the reproductive toxicity of methylparaben including on hormone activity, whether this disturbance can be modulated by baicalin is unclear. This study therefore aimed to investigate the modulatory role of baicalin on male reproductive hormone and enzyme disturbances induced by exposure to methylparaben.

## 2 Materials and methods

### 2.1 Study location

This study was carried out at the Animal House of the Department of Anatomy, College of Medicine, University of Nigeria Enugu Campus, Enugu, Nigeria.

### 2.2 Methylparaben

We obtained Methylparaben (CAS 99-76-3; EC 202-785-7; **Purity:** > 99 %), produced by Sharon Laboratories Limited, Israel, from Ejis Chemicals, 3960, Ikorodu Road, Kosofe, Lagos, Nigeria.

### 2.3 Baicalin

We obtained Baicalin (**Purity:** 85 %), produced by Botany Biosciences, USA, from Ossy Stores, Lagos, Nigeria.

### 2.4 Animals

A total of forty-five (45) adult male Wistar rats were used in this study, sourced from the Animal House of the Department of Anatomy, University of Nigeria, Enugu Campus. The rats were randomly assigned to nine (9) groups, with five (5) rats per group. They were housed in clean cages at room temperature (25–32 °C) under a 12-h light/dark cycle in the Animal House of the Department of Anatomy, University of Nigeria, Enugu Campus, following standard environmental conditions. The rats had ad libitum access to laboratory chow and drinking water, and were given two weeks to acclimatize to their new environment and diet before the study began.

### 2.5 Animal treatment

• Group 1: (Normal control); 1 mL/kg bw of distilled water for 28 days
• Group 2: (Vehicle Control); 1 mL/kg bw of peanut oil for 28 days
• Group 3: 1000 mg/kg/bw of Methylparaben only (dissolved in peanut oil) for 28 Days
• Group 4: (Low Dose Baicalin) Baicalin only 50 mg/kg/bw for 28 Days
• Group 5: (Medium Dose Baicalin) Baicalin only100mg/kg/bw for 28 Days
• Group 6: (High Dose Baicalin) Baicalin only 200 mg/kg/bw for 28 Days
• Group 7: Protective (Low Dose) Baicalin 50 mg/kg/bw + Methylparaben 1000 mg/kg/bw for 28 Days
• Group 8: Protective (Medium Dose) Baicalin 100 mg/kg/bw + Methylparaben 1000 mg/kg/bw for 28 Days
• Group 9: Protective (High Dose) Baicalin 200 mg/kg/bw + Methylparaben 1000 mg/kg/bw for 28 Days

All treatments were administered via gastric gavage, once daily. The doses of baicalin were determined based on previous research [25], which confirmed its efficacy and safety within the specified range, while the dosage of methylparaben was also informed by earlier study [13], which demonstrated that comparable concentrations could induce measurable reproductive toxicity. At the end of the experiment, the animals were anesthetized using ketamine (80 mg/kg) and xylazine (10 mg/kg) intraperitoneally, and euthanasia was performed under deep anesthesia using an overdose of sodium pentobarbital (150 mg/kg, i.p.), in accordance with the AVMA Guidelines for the Euthanasia of Animals (2020). All efforts were made to minimize pain and distress. Blood samples were collected from the heart by cardiac puncture using a needle and syringe into sample collection bottles. The blood samples were subsequently centrifuged to separate the serum for biochemical analysis.

Doses of baicalin (50, 100, and 200 mg/kg of body weight) were chosen according to the results of previous preclinical studies that have shown the pharmacological efficacy and safety of the drug in the range of doses in the rodent model of endocrine and oxidative stress-related disorders [25], [29]. The dose of methylparaben (1000 mg/kg of body weight) was selected based on previous toxicological studies [15], [30], where it produced reliable reproductive toxicity, which could be measured after 28 days of exposure. Although these doses are above normal human exposures, they are typical doses in the short-term animal models to achieve definite toxicological effects to evaluate mechanisms.

The experimental procedures involving animals and their care were performed in compliance with international, national, and institutional guidelines for the care and use of laboratory animals in biomedical research. All experimental procedures involving animals were conducted in compliance with institutional and international guidelines for animal research and welfare. Ethical approval was obtained from the Ethical Committee of the Faculty of Basic Medical Sciences, College of Medicine, University of Nigeria, Enugu, which functions as an Institutional Animal Care and Use Committee (IACUC-equivalent) and also ensure compliance to ARRIVE 2.0 guidelines [31]. The study was approved under protocol number NHREC/05/01/200BB-FWA000245B-1RB00002323.

## 3 Biochemical assays

### 3.1 Enzyme-linked immunosorbent assays (ELISA) for FSH, LH, and testosterone

Follicle-stimulating hormone (FSH), luteinizing hormone (LH), and testosterone serum levels were determined by commercial ELISA kits (MyBioSource Inc., San Diego, USA). The kits used were; FSH (Catalog No: MBS2021901), LH (Catalog No: MBS764675), and testosterone (Catalog No: MBS282195).

Each of the assays was performed in accordance with the instructions of the manufacturer, and the general procedure was the same:

Coated wells were inserted into a holder and 25–50 µL of standards, controls or serum samples were pipetted into corresponding wells. The respective hormone-specific enzyme conjugate was added to each well and the reaction incubated at room temperature (20–25 ^0^C) for 60 min. The wells were washed thrice with 300 µL of 1 × wash buffer and 100 µL of tetramethylbenzidine (TMB) substrate added. The reaction was stopped after 15 min incubation in darkness by adding 50 µL of stop solution. Absorbance was measured at 450 nm in a microplate reader, 15 min after the addition of the stop solution. Standard curves were plotted on each assay and hormone concentrations were determined thereon.

### 3.2 Assay of 17β-hydroxysteroid dehydrogenase 3 enzyme

The 17β-hydroxysteroid dehydrogenase 3 (17β-HSD3) assay is based on the method described by Marcus and Talalay [32]. This assay measures the reaction velocity as an increase in absorbance at 340 nm, resulting from the reduction of nicotinamide adenine dinucleotide (NAD). One unit of enzyme activity is defined as the reduction of one micromole of NAD per minute at 25 °C and pH 9.0, using androsterone or testosterone as a substrate.

Briefly, the spectrophotometer was adjusted to 340 nm and 25 °C. Each cuvette contained 0.6 mL of 0.166 M sodium pyrophosphate, 0.2 mL of 0.0043 M NAD, 2.0 mL of reagent-grade water, and 0.1 mL of enzyme. The mixture was incubated in the spectrophotometer for 3–4 min to achieve temperature equilibration and establish a blank rate, if any. At zero time, 0.01 mL of testosterone solution was added for 17β-hydroxysteroid dehydrogenase, and the change in absorbance at 340 nm (ΔA340) was recorded for 3–4 min. The ΔA340 per minute was calculated from the initial linear portion of the curve. This procedure was repeated using androsterone as a substrate for Δ5,3β-hydroxysteroid dehydrogenase.

### 3.3 Statistical analysis

All quantitative data was analyzed using GenStat software for Windows (Release 17.1). One-way analysis of variance (ANOVA) was used to compare the mean differences. P-value less than 0.05 (p < 0.05) was considered to be statistically significant, followed by post hoc analyses using Tukey’s multiple comparisons test for the tests that were found to be significantly different across the tables

## 4 Results

Our initial hypothesis suggested that exposure to methylparaben disrupts the HPG axis, resulting in decreased serum levels of essential reproductive hormones, thereby indicating impaired reproductive function. We evaluated the endocrine effects of methylparaben and the protective role of baicalin by measuring serum levels of key reproductive hormones and enzymatic activity through one-way ANOVA. The subsequent findings were noted.

### 4.1 Follicle stimulating hormone (FSH)

**Fig. 1:**
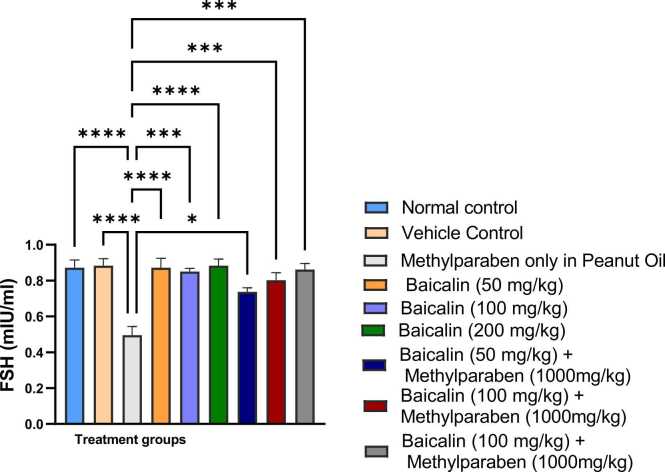
Mean serum levels of FSH of adult male wistar rats between the various groups. Values are represented as mean ± SEM. (*, **, ***, **** are significant level of difference at p < 0.05, p < 0.01, p < 0.001 and p < 0.0001).

The data presented in Fig. 1 indicate that the mean serum FSH level in Group 3 (methylparaben-only) was significantly lower (p < 0.001) when compared to rest of the other groups 1, 2, 4–9. No significant differences were noted among Groups 1, 2, 4, and 6, while Groups 5, 8, and 9 formed a statistically similar cluster. The results indicate that exposure to methylparaben correlates with a significant decrease in FSH levels, whereas baicalin at certain doses may counteract this reduction.

### 4.2 Luteinizing hormone (LH)

**Fig. 2:**
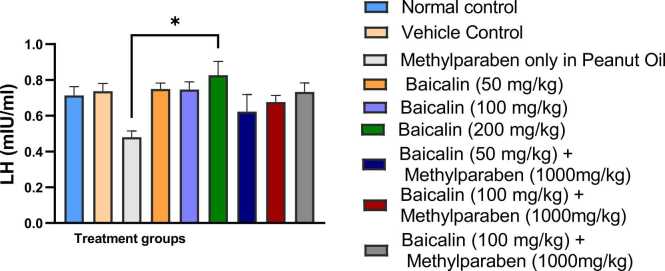
Mean serum levels Of LH of adult male wistar rats between the various groups. Values are represented as mean ± SEM. (*, **, ***, **** are significant level of difference at p < 0.05, p < 0.01, p < 0.001 and p < 0.0001).

Fig. 2 indicates a significant reduction in the mean LH level in Group 3 (p < 0.019) when compared to all other groups. Conversely, LH levels in Groups 1, 2, 4, 5, 8, and 9 exhibited statistical similarity, indicating that the treatments in these groups preserved LH levels within a similar range.

### 4.3 Testosterone

**Fig. 3:**
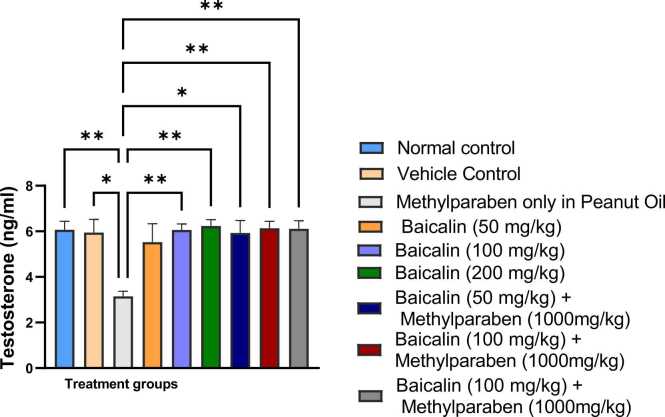
Mean serum levels of testosterone of adult male wistar rats between the various groups. Values are represented as mean ± SEM. (*, **, ***, **** are significant level of difference at p < 0.05, p < 0.01, p < 0.001 and p < 0.0001).

Serum testosterone data from Fig. 3 shows that Group 3 mean testosterone concentration remained significantly lower (p < 0.003) compared to all other groups. The observed decline is consistent with trend of endocrine disruption effects previously studied in the methylparaben-only group.

### 4.4 17β-hydroxysteroid dehydrogenase (17β-HSD)

**Fig. 4:**
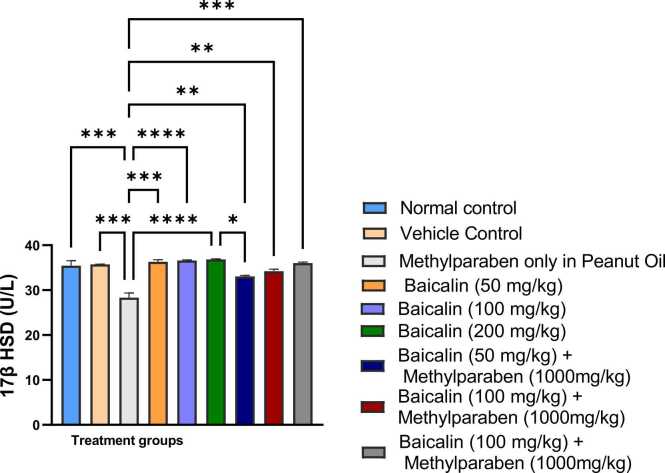
Mean serum levels of 17β-HSD3 of adult male wistar rats between the various groups. Values are represented as mean ± SEM. (*, **, ***, **** are significant level of difference at p < 0.05, p < 0.01, p < 0.001 and p < 0.0001).

Fig. 4 demonstrates that the average 17β-HSD activity in Group 3 was significantly reduced (p < 0.001) compared to Groups 1 and 2. Additionally, the 17β-HSD activity in Groups 4, 5–9 was significantly reduced in comparison to Group 3, with no notable difference observed between Groups 4 and 9.

The results indicate that exposure to methylparaben (Group 3) is linked to significant decreases in serum levels of FSH, LH, and testosterone, along with changes in 17β-HSD activity. Serum assays act as systemic indicators of hormonal function, correlating with the status of reproductive organs. The observed differences among groups indicate that baicalin treatment, given at different doses, may influence these endocrine disruptions.

## 5 Discussion

Methylparaben (MP) is the most widely used preservative in cosmetics and pharmaceutical preparations and has recently been the focus of concern based on its proposed endocrine-disrupting ability. MP studies show that it can affect hormonal regulation and reinforcement of follicular and reproductive health [17], [18]. The effects of baicalin, on MP-induced reproductive toxicity, on the hormonal and enzymatic parameters of male Wistar rats were investigated in this study. MP exposure is linked to major disruptions to the hypothalamic-pituitary-gonadal (HPG) axis, which controls reproductive function. The current study shows that the observed decreases in Follicle Stimulating Hormone (FSH), Luteinizing Hormone (LH), Testosterone (TST), and 17β-Hydroxysteroid Dehydrogenase 3 (17β-HSD3) suggest a systemic endocrine dysregulation. The characteristics of our findings are consistent with the studies [33] that show that exposure to methylparaben was linked to diminished testosterone and gonadotropins levels, probably because it disrupted the steroidogenic feedback loop. The effects of methylparaben exposure at ecologically relevant levels on neuronal health in adult zebrafish were investigated by Hu et al. [8]. Notable deficits in neuronal structure and function were revealed by the results and so place methylparaben in a potentially neurotoxic region. Apparently, oxidative stress and changes in neural signaling channels produce the noted disturbance. This indicates a wider potential risk of neurotoxic effects on aquatic organisms and, possibly, humans who are exposed to environmental parabens. Jensen et al. [34] looked at the effect of prenatal paraben exposure on anogenital distance and reproductive hormone levels during mini-puberty in subjects in the Odense Child Cohort. Results from the research showed correlations between paraben exposure and modifications in reproductive development indicators, for example, a decrease in anogenital distance and changes in reproductive hormone levels. Major concerns were exposed about parabens' endocrine disruptive properties at the most pivotal stages of development: prenatal and early childhood. Methylparaben was found to have oestrogenic characteristics that inhibit enzymes required for the biosynthesis of androgens, and thus disrupt this delicate hormonal balance necessary for healthy male reproductive integrity.

### 5.1 Role of testosterone in male reproductive health

The role of testosterone in male reproductive health is very important. The development of male reproductive tissues, such as testes and prostate, depend on it. Testosterone also helps in the production of sperm and regulates libido and sexual function. Optimal testosterone levels are important for the overall reproductive health and wellbeing of the male. Testosterone plays a pivotal role in male reproductive physiology allowing for the development and operational processes of the male reproductive system. Besides being necessary for the development of secondary sexual characteristics, testosterone is essential in regulating spermatogenesis by its action upon Sertoli cells, acting via follicle-stimulating hormone (FSH) and androgen receptors. According to the findings of Nasiri et al. [35], the synthesis of testosterone is regulated by the hypothalamic-pituitary-gonadal (HPG) axis. In this process, gonadotropin-releasing hormone (GnRH) produced by the hypothalamus prompts the anterior pituitary gland to secrete luteinizing hormone (LH) and follicle-stimulating hormone (FSH). These hormones stimulate the Leydig cells of the testes to produce testosterone. Groups exposed to methylparaben may show reduced testosterone levels, which can impair male fertility, libido, and skeletal and muscle development. This underscores the fact that interrupted testosterone synthesis has very serious systemic ramifications.

### 5.2 Synthetic pathways of testosterone and their enzymatic involvement

The process of testosterone synthesis consists of several enzymatic reactions with initial and rate-limiting steps, emphasizing its complexity. Transport of cholesterol into mitochondria is critical and dependent on the protein Steroidogenic Acute Regulatory (StAR). Cholesterol is converted within this organelle by CYP11A1 to pregnenolone, the process of side chain cleavage enzyme. After this, androstenedione is formed by a series of enzymatic reactions and is further converted to testosterone by 17β-HSD3, the last and the slowest enzyme in the pathway [36]. According to Jensen et al. [34], the disruption of this pathway by methylparaben may originate from its effects on StAR protein expression and 17 β-HSD3 activity. This interference decreases testosterone bioavailability due to endocrine distributing chemicals and demonstrates how sensitive the enzymatic machinery is to them.

### 5.3 Mechanisms of methylparaben-induced toxicity

MP’s ability to mimic estrogen by binding to estrogen receptors is a key mechanism of its endocrine-disrupting action. This interaction can alter normal hormonal signaling, leading to disruptions in the hypothalamic-pituitary-gonadal (HPG) axis and consequent reproductive dysfunctions [12], [33]. The significant reduction of follicle stimulating hormone (FSH), luteinizing hormone (LH), testosterone, and 17β hydroxysteroid dehydrogenase (17β-HSD) in these animals agreed with the deleterious effect of MP treated animals on male reproductive hormones. Additionally, MP has been implicated for oxidative stress and mitochondrial dysfunction, which concur to negatively alter reproductive tissues [13]. It is shown that MP exposure alters mitochondrial bioenergetics and depletes the enzymatic antioxidant defense and causes cellular damage [8], [14]. This is consistent with the observed decrease in enzymatic activity and hormonal profiles of group 3 rats on MP.

### 5.4 Methylparaben’s disruptive effects on reproductive hormones

Methylparaben has been widely documented as an endocrine disruptor known to disrupt the HPG axis. We hypothesize that its estrogenic activity functions via estrogen receptors and acts on a negative feedback to inhibit GnRH release and downstream gonadotropin secretion [37], [38]. This mechanism might explain the marked reduction in FSH, LH and testosterone seen in this study. Also, analyses of transcription by Hu et al. [8], indicate that methylparaben disrupts steroidogenic gene expression, including those of the StAR protein and 17β-HSD3. These data support compelling evidence that methylparaben has endocrine-disrupting properties that go beyond estrogenic action to include perturbations in androgen biosynthesis and regulation.

### 5.5 Evidence of methylparaben's endocrine-disrupting effects

Many studies have shown that methylparaben exposure disrupts reproductive outcomes. Smith et al. [37], show that parabens, including methylparaben, lower serum testosterone levels and impair spermatogenesis in experimental models. As demonstrated by Aker et al. [38], methylparaben may be a potential suppressor of LH and FSH secretion so as to disrupt the hormones that allow male fertility. Our findings are consistent with the studies that showed that methylparaben not only decreased gonadotropin levels but also directly inhibited 17β-HSD3, the essential enzyme in testosterone biosynthesis. Methylparaben-induced reproductive toxicity is secondary to two of these mechanisms and signifies the extent to which methylparaben hinders reproductive health.

### 5.6 Protective role of baicalin against hormonal disruption

A natural flavonoid, baicalin, has been suggested as a protective agent against the side effects of endocrine disruptors. Thus, in the present study, the administration of baicalin to groups exposed to methylparaben normalized levels of FSH, LH, and testosterone, indicating its potential to ameliorate hormonal disruptions. According to Akilah et al. [29], baicalin possesses properties that combat oxidation and inflammation, which may help mitigate the stress induced by methylparaben. This also agrees with Martins et al. [30], finding that oxidative stress is a major pathway by which methylparaben induces toxic effects. It seems likely that baicalin scavenges reactive oxygen species and upregulates steroidogenic enzymes to restore hormonal homeostasis. 17β-HSD3’s role on testosterone synthesis and Methylparaben inhibition of 17β-HSD3 activity. In the steroidogenic pathway, 17β-HSD3 is an essential enzyme in testosterone formation through conversion of androstenedione and as the last and rate limiting step. Sobel and Imperato-McGinley [39] highlight that, in the event of disruption of 17β-HSD3 activity, this can cause a great reduction in testosterone biosynthesis which results in hypogonadism and infertility. The inhibition of 17β-HSD3 by Methylparaben seen in this study further shows that the enzyme is susceptible to environmental endocrine disruptors. The inhibition is in accordance with Engeli et al. [33], that parabens can interfere with androgen and estrogen biosynthesis 17β-HSD enzyme activity, leading to disruption. This study provides a mechanistic explanation for the reduced testosterone levels by inhibition of 17β-HSD3 by methylparaben. Parabens can act as competitive inhibitors of 17β-HSD enzymes to disrupt the enzymatic conversion of precursors into active hormones [40]. Not only does this mechanism impair testosterone biosynthesis but as a result there are also downstream effects on spermatogenesis and the male reproductive capacity.

### 5.7 Reproductive health and endocrine-disrupting compounds

Methylparaben and other endocrine-disrupting compounds (EDC) are known to interfere with hormone biosynthesis, secretion, and metabolism. These compounds frequently act by mimicking or blocking endogenous (transitioned to hormone) hormones, interfering with receptor signaling or enzymatic activity [41], [42]. Martins et al. [30], reported that oxidative stress induced by methylparaben disrupts hormonal balances, resulting in systematic imbalances. These findings have implications beyond variation in individual reproductive health to population-level fertility trends, as parabens are now widely used in a variety of consumer products.

### 5.8 Baicalin’s protective mechanisms against methylparaben toxicity

Baicalin’s ability to restore 17β-HSD3 activity and normalize testosterone levels highlights its therapeutic potential. Its antioxidant properties may mitigate oxidative stress-induced damage to Leydig cells, thereby preserving the integrity of steroidogenic pathways. The studies of Akilah et al. [29], and Martins et al. [30], demonstrate the potential of baicalin to help in the cellular resistance against endocrine disruptors and therefore, can serve as a future therapeutic target.

### 5.9 Potential future research directions

Future research of baicalin's protective mechanism against reproductive toxicity from methylparaben exposure must include in vivo investigations using animal models including Wistar rats alongside guinea pigs and non-human primates and mice for investigating pharmacodynamic effects. Translational studies that employ these models should investigate the pharmacokinetic behavior and bioavailability of baicalin and establish its comprehensive safety profile when used under prolonged exposures in order to close the gap to human medical use. The tissue-level evaluation of reproductive organs combined with the molecular analysis and histopathological investigation would give valuable insights about baicalin's mechanisms of action. The technological methods of RT-qPCR and RNA-sequencing should be implemented to investigate how baicalin modifies gene expression levels in the pathways of the hypothalamic-pituitary-gonadal (HPG) axis and steroidogenesis and oxidative stress for molecular target identification. Studies involving multiple endocrine-disrupting chemicals as found in environmental exposures will allow researchers to test baicalin's actual effectiveness in toxicological situations. Future research should focus on developing better baicalin formulations to increase its substance absorption as well as conducting human clinical trials that evaluate its treatment effects and security in people who frequently encounter parabens.

### 5.10 Potential limitations of the study

Numerous constraints limit the potential of the research project despite its existing positive features. The study's findings are not broadly applicable to multiple species or different genders since researchers worked with only male Wistar rats. Scientific data on human reproductive physiology together with hormonal regulation and flavonoid supplementation outcomes show major variations which require scientists to exercise caution when drawing connections. The research period of 28 days might not capture the complete endocrine and reproductive effects on both methylparaben exposure and baicalin supplementation during regular and prolonged treatment. This research mainly uses serum biomarkers instead of performing direct tissue analyses or gene expression studies which might have provided more precise cellular-level reproductive tissue data. Also, the mechanism by which baicalin modulates the hormonal axis—whether via receptor binding, enzyme modulation, or antioxidant pathways—was not fully elucidated and requires deeper biochemical and molecular exploration. Finally, as baicalin’s bioavailability and metabolism in humans are not well understood, its potential interactions with human drug-metabolizing enzymes or endogenous hormones remain speculative and merit further pharmacological scrutiny.

The relative small sample size of five animals per group is another limitation of the present study. Although this sample is consistent with preliminary toxicological tests and corresponds to ethical concerns of limiting the animal population, the number can be a drawback to the statistical strength of the study, as well as the ability to generalize the results. Further research with bigger groups is justified in order to validate these findings and increase the strength of statistical conclusions.

Additionally, the study relied solely on serum hormonal levels and 17β-HSD3 enzymatic activity as endpoints. The lack of histopathological examination of reproductive tissues along with molecular measurements, e.g. gene expression profiling of major steroidogenic enzymes or oxidative stress markers, limits the mechanistic interpretation of our results. Future studies should include these tissue-level and molecular endpoints to give a more direct indication of cellular and pathway-specific protective responses of baicalin.

## 6 Conclusion

Substantial decreases in FSH, LH, and testosterone levels are observed in this study indicative of a reproductive toxicity of methylparaben. As a natural medicinal agent, Baicalin has the potential to exhibit protective benefits due to its antioxidant properties and its regulation of 17β-HSD3 activity. Our results highlight the importance of further understanding natural substances, like baicalin, that prevent the adverse effects caused by endocrine disruptors, given their widespread use as stabilizers in consumer goods. Further research will need to clarify the molecular mechanisms through which baicalin confers its protective properties and determine more broadly whether baicalin has the potential as a therapeutic for reproductive health.

## CRediT authorship contribution statement

**Daniel Itiza Akaahan Daniel Itiza:** Writing – original draft, Methodology, Investigation, Funding acquisition, Conceptualization. **Augustine Uche Agu:** Visualization, Software, Methodology, Funding acquisition, Formal analysis. **Anyanwu Emeka Godson:** Writing – review & editing, Validation, Supervision, Project administration, Conceptualization. **Nkemjika Chinyere Anyanwu:** Writing – review & editing, Resources, Project administration, Formal analysis, Data curation. **Vivian Onyinye Orjiako:** Project administration, Methodology, Data curation, Conceptualization.

## Ethical Approval

All experimental procedures involving animals were conducted in accordance with the guidelines and approval of the Ethical Committee of the Faculty of Basic Medical Sciences, College of Medicine University of Nigeria Enugu with ethical approval number NHREC/05/01/200BB-FWA000245B-1RB00002323.

## Statement of Informed Consent

This study involved only animal experiments; thus, informed consent from human subjects was not applicable.

## Funding Statement

This work was self-sponsored.

## Statement of Human and Animal Rights

All procedures performed in this study involving animals comply with the ethical standards of the institution and adhere to the principles outlined in the 1964 Helsinki Declaration and its later amendments.

## Conflict of Interest Statement

The authors declare that there is no conflict of interest regarding the publication of this paper.

## Declaration of Competing Interest

The authors declare that they have no known competing financial interests or personal relationships that could have appeared to influence the work reported in this paper.

## Data Availability

Data will be made available on request.
